# Latitude, sun exposure and vitamin D supplementation: associations with quality of life and disease outcomes in a large international cohort of people with multiple sclerosis

**DOI:** 10.1186/s12883-015-0394-1

**Published:** 2015-08-05

**Authors:** George A. Jelinek, Claudia H. Marck, Tracey J. Weiland, Naresh Pereira, Dania M. van der Meer, Emily J. Hadgkiss

**Affiliations:** Neuroepidemiology Unit, Melbourne School of Population and Global Health, The University of Melbourne, Melbourne, VIC Australia; Emergency Practice Innovation Centre, St Vincent’s Hospital, Melbourne, VIC Australia; Emergency Department, Box Hill Hospital, Box Hill, VIC Australia

## Abstract

**Background:**

A growing evidence base implicates vitamin D, sun exposure and latitude in the aetiology of multiple sclerosis (MS), however there are less data on the associations of these variables with disease outcomes.

**Methods:**

We undertook a cross-sectional survey of over 2000 people with MS recruited through internet platforms, seeking self-reported data on geographical location, intentional sun exposure for health, and supplementation with vitamin D, among other lifestyle variables. We also requested data on health-related quality of life (MSQOL-54), self-reported doctor-diagnosed relapse rate, and disability (Patient Determined Disease Steps). Bivariate and multivariate analyses were used for comparisons, including multiple linear regression modeling.

**Results:**

Of 2301 participants, 82.3 % were female, median age was 45 years (IQR 38–53 years), with a median time since diagnosis of 6 years (IQR 3-12 years), the majority (61.6 %) having relapsing-remitting MS. Nearly two-thirds (64.6 %) lived in the Northern hemisphere, mostly in developed countries. Most (66.8 %) reported deliberate sun exposure to raise their vitamin D level, and the vast majority (81.8 %) took vitamin D supplements, mostly 2000–5000 IU a day on average. Unadjusted regression modeling incorporating deliberate sun exposure, latitude and vitamin D supplementation showed strong associations of sun exposure with HRQOL which disappeared when controlling for gender, age, disability, physical activity, and fish consumption. In contrast, associations between vitamin D supplementation and HRQOL were maintained adjusting for these variables, with a dose–response effect. Only latitude had significant adjusted associations with disability, with an increase of latitude by one degree (further from the equator) predicting increased odds of moderate disability (OR 1.02 (95 % CI 1.01–1.04)) or high disability (OR 1.03 (95 % CI 1.01–1.05)) compared to no/mild disability. Similarly, latitude was related to relapse rate, with increase in latitude of 1 degree associated with increased odds of having more relapses over the previous year (1.01 (1.00–1.02)).

**Conclusions:**

We detected significant associations between latitude, deliberate sun exposure and vitamin D supplementation and health outcomes of this large group of people with MS. Vitamin D is likely to have a key role in these associations and its role in the health outcomes of people with MS urgently requires further study.

**Electronic supplementary material:**

The online version of this article (doi:10.1186/s12883-015-0394-1) contains supplementary material, which is available to authorized users.

## Background

Multiple sclerosis (MS) is a progressive neurodegenerative disease with a complex aetiology. MS is a familial disease, and genetic background plays a significant role in disease development, although of lesser importance than environmental and lifestyle factors [1–3]. In contrast, for disease progression or severity, no genetic markers have been demonstrated to date [4]. This highlights the potentially important role of risk factor modification as part of a secondary preventive approach to disease management.

Previous research has examined potential environmental and lifestyle risk factors for MS disease development and progression. The longest studied of these factors has been the association of increasing latitude with MS incidence [5, 6]. A substantial body of research has linked this association to reduced sun exposure, probably largely through low exposure to ultraviolet light mediated by reduced endogenous vitamin D production [7]. Less attention has been focused on the effects of sun exposure on MS disease course, although clinical trials are now suggesting vitamin D supplementation has a favourable effect. Some are suggesting that vitamin D supplementation should now be considered standard of care for people with MS (PwMS) [8]. While opinion leaders in neurology have publicly stated that if they themselves were diagnosed with early demyelination, they would take high dose vitamin D supplements, they still debate whether the evidence of benefit is sufficiently strong to warrant routine supplementation for their patients, and if so, at what dosage [9]. The effects of sun exposure and vitamin D supplementation on MS disease progression are biologically plausible, given their known immunomodulatory effects in the context of the central role of immune dysfunction in MS [10].

Through Web 2.0 platforms, we have assembled a large cohort of PwMS from 57 countries for the purpose of examining associations between environmental and lifestyle factors and measures of quality of life and disease outcomes. This cohort is somewhat unique in that it contains a large proportion of PwMS actively modifying lifestyle factors thought to be associated with MS disease course, enabling robust comparisons between those who do and do not modify such factors [11]. If sun exposure and vitamin D supplementation have the beneficial effects postulated in research to date, we would expect to see associations in this cohort between the latitude of their place of residence and these outcomes; we would also expect to see a signal of reduced relapse rate and disease progression with vitamin D supplementation. This study aimed to test these associations.

## Methods

### Participants and data collection

The methodology of this study has previously been described in some detail [11]. Briefly, using the online software SurveyMonkey®, we developed a webpage describing the study aims and inviting PwMS to take part. We posted a link to this webpage on websites, blogs, forums, and social media in which the principal investigator (GJ) was actively involved, including his own website (www.overcomingms.org; ‘OMS’). Online groups and pages used by PwMS with over 500 members or followers were targeted. Several follow up notices were posted over the 15 week recruitment period. MS societies were also asked to circulate details to their members.

After reading a participant information sheet, responders were advised that participation in the survey would constitute consent to the study. Inclusion criteria were adults of 18 years of age or more, who had been formally diagnosed with MS by a medical doctor and who could undertake an English language survey. We also sought contact details to enable planned longitudinal follow up. Data were stored in re-identifiable format, and access was restricted to members of the research team. Ethical approval was granted by St Vincent’s Hospital Melbourne Human Research Ethics Committee (LRR 055/12).

### Data collected and tools used

Overall, the survey consisted of 163 questions, and took approximately 40 min to complete. Validated tools were used where possible, although for several variables, tools had not previously been validated. Socio-demographic data included age, gender, current location of residence, country of birth, cultural background, marital status, number of children, employment, education, height and weight. Disease-specific data included whether MS diagnosis had been confirmed by a medical doctor, year of diagnosis, first year of symptoms, diagnostic investigations undertaken, type of MS on diagnosis and currently. Participants were asked with a researcher-devised question how many physician-diagnosed relapses they had had in the previous 12 months and the last 5 years. Level of disability was assessed using the Patient-Determined Disease Steps (PDDS), a validated self-reported surrogate tool to the Expanded Disability Status Scale (EDSS) commonly used by neurologists to assess gait disability [12]. PDDS is scored ordinally from 0 (normal) to 8 (bed bound).

Health-related quality of life (HRQOL) was assessed with the validated and widely used Multiple Sclerosis Quality Of Life-54 (MSQOL-54), developed from the RAND 36-Item Health Survey (SF-36) and supplemented with 18 additional items. MSQOL-54 consists of 52 items in 12 scales, and two single items, producing two composite scores – the physical and mental health composites [13]. Whether participants deliberately exposed themselves to the sun to increase vitamin D production (yes/no) and frequency and dosage of vitamin D supplementation were recorded using researcher-developed questions, as there were no validated tools for these data. Vitamin D supplementation dose in International Units (IU) (0, 1000, 2000, 3000, 4000, 5000, 6000, 7000, 8000, 9000, 10,000, 20,000, 30,000, 40,000, 50,000, 100,000, 250,000, 500,000) and frequency (daily, every second day, weekly, monthly) gave rise to an average daily vitamin D supplementation dose which was collapsed into four categories for data analysis purposes (none, 1–2000 IU, 2001–5000 IU and >5000 IU). Latitude (degrees and minutes) was obtained from the city and country of residence.

### Data analysis

Data were analysed using IBM SPSS Statistics 22.0 (IBM Corporation). Continuous data were summarised using mean (95 % CI) or median (interquartile range (IQR)) and categorical data using number (N) and percentage. Comparisons involving two continuous variables were undertaken using independent samples Kruskal-Wallis test as the variables latitude and relapse rate were not normally distributed, with separate Mann Whitney U tests for post-hoc analyses. For categorical data Pearson’s Chi Square was used, with assessment of adjusted standardised residuals to indicate under- or over-representation of groups. For all inferential tests, two-tailed tests of significance were used and the criterion for significance was set at .05.

Linear regression was used to predict the Physical Health and Mental Health composite scores and the Health Perception and Energy scale scores of the MSQOL-54 using the three variables of interest: latitude, deliberate sun exposure, and average daily vitamin D supplementation. First these predictor variables were entered separately to obtain crude odds ratios (and 95 % CI), and then together in a multiple linear regression model including the variables age, gender, disability, fish consumption, and physical activity to assess adjusted odds ratios (95 %CI). Relatively stable factors including gender, age, disability and fish consumption were included in the regression models as these can impact on the variables of interest. Physical activity was controlled for as many people exercise outdoors and it may therefore impact on sun exposure and vitamin D levels. Multinomial logistic regression was used to predict disability in a model including the variables gender, age, fish consumption and physical activity. Ordinal logistic regression was used to predict relapse rate in a model including the variables disability, gender, age, fish consumption and physical activity. Testing of assumptions for regression analysis were performed, including an examination of multi-collinearity to ensure that continuous independent variables were not closely correlated (having a bivariate correlation >0.70). All percentages reported were adjusted for missing data (due to item non-completion) on an item by item basis.

## Results

### Demographics

As previously reported, 2469 people with a self-reported doctor diagnosis of MS commenced the survey ^11^. The sample described here, answering questions regarding location of residence, and with a self-reported doctor diagnosis of MS consisted of 2466, with varying completion for other individual items. 1894 (82.3 %) women and 407 (17.7 %) men with MS with a median age of 45 years (IQR 38–53 years), had been diagnosed a median of 6 years prior (IQR 3–12) and most had relapsing-remitting MS (1490, 61.6 %). Many worked full time (799, 32.5 %) or part time (524, 21.3 %) and a significant proportion were retired due to medical reasons or disability (569, 23.2 %). Most lived in the United States (807, 32.7 %), Australia (627, 25.4 %) or the United Kingdom (417, 16.9 %), although 57 countries were represented in total.

### Latitude, deliberate sun exposure, and vitamin D supplementation

Totals of 1592 (64.6 %) and 874 (35.4 %) lived in the northern and southern hemisphere respectively. Most people (nearly half) lived between latitudes 30 and 40 degrees, or for those in the US, between New Orleans and Columbus, Ohio. The median latitude was 39 degrees and 83 min (IQR 34.92–48.66). This is approximately the latitude of Philadelphia in the Northern Hemisphere, or King Island in the Bass Strait between Australia and Tasmania, in the Southern Hemisphere. A total of 1504 (66.8 %) reported that they intentionally got sun exposure to raise their vitamin D level. The majority took vitamin D supplementation (1794, 81.8 %), mostly between 2000 and 5000 IU on average daily (Table 1).

**Table 1:** Average daily dose of supplemental vitamin D (self reported)

Vitamin D dose	N	Percent
>5000 IU	447	20.4
2001–5000 IU	734	33.5
1–2000 IU	613	28.0
None	399	18.2

Latitude was not related to vitamin D supplementation, or deliberate sun exposure. Vitamin D supplementation was associated with intentional sun exposure (*P* < .001) with those intentionally getting sun exposure more likely to be taking larger doses (over 2000 IU on average per day), and those not intentionally getting sun exposure more likely to be taking lower doses (2000 IU or less per day).

### Quality of life

Additional file 1 displays the results of a regression model incorporating deliberate sun exposure, latitude and vitamin D supplementation individually with HRQOL outcomes as well as a regression model incorporating these variables together with gender, age, disability, physical activity, and fish consumption (adjusted). The apparent quality of life associations of deliberate sun exposure appear to be strongly influenced by the variables being adjusted for, such that they disappear when the regression model is adjusted for these variables. In contrast, the apparent associations of vitamin D supplementation are generally maintained, although diminished, on adjusting for these variables, with a dose–response effect and quality of life differences of clinically significant magnitude. MSQOL-54 measurements are derived from the SF-36 and it is generally accepted that a five point increment in this scale is clinically significant [14, 15].

### Disability

Disability was related to latitude (*p* = .014) with non-parametric post hoc testing showing a significant difference in latitude between the mild disability group and the moderate disability group (*p* = .009). Median latitudes for these groups were 39.00 (34.83–46.80) for mild disability, 40.59 (35.30–50.73) for moderate and 41.26 (34.98–51.14) for the high disability group.

Those intentionally getting sun exposure were more likely to be in the mild disability group and less likely to be in the moderate or high disability groups compared to those not intentionally getting sun exposure (*p* < .001, Table 2). Vitamin D supplementation was related to disability also with those taking 2001-5000 IU per day more likely to be in the mild disability group, and less likely to be in high disability group while those taking 1–2000 IU per day were less likely to be in the mild disability group and more likely to be in the moderate disability group (*p* < .001) (Table 3).

**Table 2:** Intentional sun exposure and level of disability

Intentional sun exposure		Level of disability		
		Mild	Moderate	High
Yes	N	875^a^	491^b^	133^b^
	%	58.4	32.8	8.9
No	N	360^b^	280^a^	103^a^
	%	48.5	37.7	13.9

^a^overrepresented ^b^underrepresented

**Table 3:** Vitamin D supplementation and level of disability

Average daily vitamin D supplementation		Level of disability		
		Mild	Moderate	High
>5000 IU	N	257	138	52
	%	57.5	30.9	11.6
2001–5000 IU	N	439^a^	236	58^b^
	%	59.9	32.2	7.9
1–2000 IU	N	293^b^	243^a^	74
	%	48.0	39.8	12.1
None	N	207	140	48
	%	52.4	35.4	12.2

^a^overrepresented ^b^underrepresented

After adjusting for gender, age, physical activity, fish consumption, deliberate sun exposure, and vitamin D supplementation a logistic regression model revealed that an increase of latitude by one degree (further away from the equator) predicted an increased odds of being in a moderate disability category (OR 1.02 (95 % CI 1.01–1.04), *p* < .001) or high disability category (OR 1.03 (95 % CI 1.01–1.05), *p* = .001) compared to being in the no/mild disability category. None of the other variables of interest contributed significantly to level of disability in this model (data not shown).

### Relapse rate

Annualised relapse rate was significantly associated with vitamin D supplementation (*p* = .001) with significant differences between those taking none (mean .92) and all other groups: those taking 1–2000 IU (mean .63, *p* < .001); 2001-5000 IU (mean .61, *p* = .003); and those taking >5000 (mean .62, *p* < .001). Sensitivity analyses were undertaken comparing relapse rate according to vitamin D supplementation using non parametric tests which confirmed the findings of parametric analyses (data not shown). Differences between other groups were not significant. Relapse rate was not related to intentional sun exposure (data not shown).

Ordinal regression incorporating the variables gender, age, physical activity, fish consumption, deliberate sun exposure, latitude and vitamin D supplementation revealed that the only variable of interest to significantly predict relapse rate was latitude, with increase in latitude of 1 degree predicting a 1 % increase in the odds of having more relapses over the previous 12 months (1.01 (1.00–1.02), *p* = .049).

## Discussion

Epidemiological data over many years has confirmed the striking latitude gradient of MS incidence, both world-wide and within countries. Australia is a good example, with the incidence of MS varying around seven-fold between cities in northern versus southern Australia [16]. This has long been postulated to be due to sun exposure, and elegant Australian studies have confirmed that both recalled time in the sun, particularly winter sun during childhood, and solar skin damage relate inversely to incidence of MS [17]. There are less data about a relationship between latitude and MS disease course. However, recent work from the MSBase international registry examining 32,762 relapses from 9811 patients across 30 countries from both hemispheres found a strong seasonal variation in onset of relapses with peaks in early spring (after a period of decreased sun exposure) and troughs in autumn in both hemispheres [18]. This confirmed a prior meta-analysis with similar findings from the northern hemisphere [19].

There has been a long delay in testing the effects of vitamin D supplementation on disease course in MS, presumably due to little commercial incentive to test this non-patentable naturally occurring agent, however a number of large international studies are now underway to examine this issue. Trials to date suggest a benefit, with markedly reduced conversion of optic neuritis to MS [20] and fewer lesions when added to existing disease-modifying medication [21]. While the effect is biologically plausible, it is possible that higher serum vitamin D levels are just a marker of increased sun exposure, and it is both the direct effect, and other as yet unknown indirect effects of sun exposure that mediate beneficial immunomodulatory improvements in disease course for PwMS [22, 23].

We sought to shed more light on whether latitude was associated with disease activity not only disease incidence, and whether we could detect a signal of a beneficial effect on disease course and other health outcomes for PwMS who supplemented with vitamin D. Our data showed that disability was indeed related to latitude with increasing disability the further away from the equator people lived, with a 2–3 % increase in odds of a higher disability category for each degree further from the equator. Similarly, there was some association between lower disability and intentional sun exposure and increasing dose of vitamin D supplementation. Taking vitamin D supplements was also associated with around a third lower annualised relapse rate, while increasing latitude was associated with a small increase in the odds of more relapses. Intentional sun exposure was not associated with relapse rate in our sample.

There were no associations between quality of life domains examined and latitude, and those associations with deliberate sun exposure were not maintained in adjusted regression modelling, suggesting a complex relationship between the variables being adjusted for and sun exposure. Vitamin D supplementation however did have clinically significant associations with these quality of life outcomes, with a dose–response effect suggesting a causal relationship. Vitamin D supplementation is known to have quality of life benefits and these associations may reflect this broader health benefit [24].

Our data suggest a complex relationship between the variables of interest of latitude, sun exposure and vitamin D supplementation, and the additional variables controlled for in our study, namely exercise levels and frequency of fish consumption. The latter two are both likely to affect serum vitamin D levels, as well as having associations with the outcome variables of interest in their own right [25, 26]. Disentangling these relationships statistically in a large sample can help shed light on the contribution of these inter-related factors related to health outcomes for PwMS, but ultimately, these questions are best answered by randomised controlled trials. For vitamin D supplementation, this should present no particular methodological problems and is long overdue. For sun exposure, trial design may be more complex, but the research no less imperative.

### Limitations

Some measures were relatively crude with a simple yes/no answer in the absence of a validated tool for sun exposure. We didn’t have an indication of amount of daily sun exposure or life time exposure, but rather whether participants deliberately exposed themselves to sun for health benefit and their current location. All measures were self-reported, and therefore may be subject to recall bias. Vitamin D supplementation was self-reported and not validated with blood tests. Average daily supplement dose was calculated and we didn’t take into account differences between those supplementing with infrequent large doses and those supplementing more frequently with lower doses.

## Conclusions

Associations were detected in our large international sample of PwMS between latitude, deliberate sun exposure and vitamin D supplementation and health outcomes including disability, relapse rate and quality of life. Vitamin D is likely to have a pivotal role in these associations; its role in MS health outcomes urgently requires detailed exploration with well-designed clinical trials.

## Additional file

Additional file 1: Table S1. Regression coefficients for health related quality of life outcomes. (DOCX 95 kb)
