# Supplementary material for: Latitude, sun exposure and vitamin D supplementation: associations with quality of life and disease outcomes in a large international cohort of people with multiple sclerosis
Source: BMC Neurol. 2015 Aug 5;15:132. doi: 10.1186/s12883-015-0394-1 (PMC4525738; doi:10.1186/s12883-015-0394-1)
Supplement: Additional file 1: Table S1. — Regression coefficients for health related quality of life outcomes. (DOCX 95 kb) [file 12883_2015_394_MOESM1_ESM.docx]

| **Additional file 1. Regression coefficients for health related quality of life outcomes** | | | | | | | | | | |
| --- | --- | --- | --- | --- | --- | --- | --- | --- | --- | --- |
|  |  | Physical health composite score | | Mental health composite score | | Energy subscore | | Health perception subscore | |  |
|  |  | Unadjusted B (95% CI) | Adjusted B (95% CI)^a^ | Unadjusted B (95% CI) | Adjusted B (95% CI)^b^ | Unadjusted B (95% CI) | Adjusted B (95% CI)^c^ | Unadjusted B (95% CI) | Adjusted B (95% CI) ^d^ |  |
| Deliberate sun exposure | No | reference | reference | reference | reference | reference | reference | reference | reference |  |
|  | Yes | **6.55*****  **(4.51-8.58)** | .59  (-1.05-2.24) | **4.70*****  **(2.80-6.60)** | 1.44  (-.49-3.37) | **6.80*****  **(4.84-8.77)** | 1.72  (-.23-3.67) | **6.47*****  **(4.48-8.45)** | 1.66  (-.31-3.63) |  |
| Latitude | Degrees and minutes | -.11  (-.22-.00) | -.01  (-.09-.08) | -.09  (-.19-.02) | -.03  (-.13-.08) | **-.12***  (-.23 to -.02) | -.05  (-.15-.06) | -**.24*****  **(-.34 to -.13)** | **-.18****  **(-.29 to -.08)** |  |
| Vitamin D supplementation | None | reference | reference | reference | reference | reference | reference | reference | reference |  |
|  | 1-2000IU | **3.08***  **(.14-6.02)** | **3.42****  **(1.12-5.71)** | **5.50*****  **(2.78-8.21)** | **3.79****  **(1.11-6.46)** | **5.04*****  **(2.22-7.87)** | **3.66****  **(.95-6.36)** | **2.87***  **(.02-5.72)** | 1.63  (-1.10-4.36) |  |
|  | 2001-5000IU | **8.03*****  **(5.22-10.84)** | **3.04****  **(.81-5.27)** | **8.11*****  **(5.48-10.74)** | **3.87****  **(1.22-6.52)** | **7.62*****  **(4.89-10.35)** | 2.64  (-.03-5.31) | **6.05*****  **(3.30-8.81)** | 1.78  (-.91-4.48) |  |
|  | >5000IU | **8.91*****  **(5.82-12.00)** | **5.10*****  **(2.66-7.53)** | **8.61*****  **(5.71-11.51)** | **5.17*****  **(2.28-8.06)** | **10.20*****  **(7.17-12.22)** | **5.74*****  **(2.81-8.67)** | **9.83*****  **(6.78-12.89)** | **6.30*****  **(3.34-9.25)** |  |
| *p<.05 **p<.01 ***p<.001 Bold typeface indicates a significant result. Other variables included in the adjusted regression model: Age, gender, disability, physical activity, and fish consumption. R squared: ^a^.466 ^b^.131 ^c^ .200 ^d^.192 | | | | | | | | | | |
